# Sustainable Work Performance: The Roles of Workplace Violence and Occupational Stress

**DOI:** 10.3390/ijerph17030912

**Published:** 2020-02-01

**Authors:** Samma Faiz Rasool, Mansi Wang, Yanping Zhang, Madeeha Samma

**Affiliations:** 1 Postdoctoral Station of Statistical, Guangzhou University, Guangzhou 510006, China; samma@gzhu.edu.cn; 2 School of Innovation and Entrepreneurship, Entrepreneurship Institute, Guangzhou University, Guangzhou 510006, China; 3 School of Management, Guangzhou University, Guangzhou 510006, China; 4 School of Management, Shanghai University, Shanghai 200444, China; madeeha@i.shu.edu.cn

**Keywords:** workplace violence, occupational stress and sustainable work performance

## Abstract

The purpose of this study is to analyze the relationships between workplace violence, occupational stress, and sustainable work performance. Multiple dimensions of workplace violence (harassment, mobbing, ostracism, and stalking) were used in this study. A questionnaire survey was used, composed of 48 items with a 5-point Likert scale (1, strongly disagree, to 5, strongly agree). Data were collected from 15 hospitals in the vicinity of Karachi, Lahore, and Islamabad, Pakistan. The target population of this study consisted of doctors, nurses, and paramedical staff. We distributed 500 questionnaires among the target population. In total, 345 usable questionnaires were returned, resulting in a response rate of 69%. Partial least squares structural equation modeling was used to test the direct and indirect effects. The results of this study highlight that in both direct and indirect relationships, workplace violence negatively influences sustainable work performance. The findings of this study are as follows: First, harassment reduces employee morale, which consistently lessens employees’ work performance. Second, mobbing at the workplace reduces productivity, increases levels of stress, anxiety, depression, and irritability, and increases low work engagement, work absences, and work destruction. Third, ostracism at the workplace reduces motivation among workers and organizations, which reduces work efficiency. Work performance is undermined due to stalking at the workplace because it creates a bad image and brings toxicity among colleagues and peers. Fourth, occupational stress is considered a stigma among employees who are facing stress at the workplace. We can conclude that if employees are happy and healthy, they can be their most productive. So, organizations need to construct a culture where employees can be at their best and shine.

## 1. Introduction

The influence of workplace violence (WV) on sustainable work performance (SWP) has been debated in previous studies [1,2,3]. However, insights into the occupational stress it creates among employees are still not discussed in the literature. Stressful events in different aspects of normal life (work, friends, and family) impose a high psychological burden, which may negatively affect people’s performance at work. Although some individuals rise to the challenges of such stresses, others try to escape from them. In the current era, many people who experience daily stress are resigned to enduring workplace violence. This paper addresses the interventional effects of occupational stress (OS) between WV and SWP to determine implications for academicians and practitioners.

Interactions among people at workplaces are apparent and may have positive or negative dimensions. Usually, positive or negative interactions between people lead to different outcomes. Sometimes they lead to a productive working environment, but in some cases, they lead to toxicity among working professionals [4]. Based on the notion that workplace violence deteriorates work performance, it also incites working hassles and stress among employees. Not only does workplace violence cause stress and hassle among employees, but it also provokes distress in organizational managers in measuring workers’ efficiency and organizational gains. To visualize the determinants of workplace violence, organizational managers put effort into re-examining their human resources (HR) practices to regain a healthy working environment and sustainable work performance. In recent years, employee turnover due to workplace violence has gained a great deal of attention among practitioners and academicians [2,5]. In eradicating WV and its effect on SWP, the extant literature presents many thought-provoking insights, yet ways to prevent and resolve toxicity remain vital for managers and researchers [6]. No doubt, workplace violence deteriorates sustainable work performance, and measures to halt such behavior need further clarification by organizational heads. Employees/workers are the backbone of any organization, but they are ignored in many sectors. A supportive environment for workers can improve their work performance, whereas WV (harassment, mobbing, ostracism, and stalking) can deteriorate sustainable work performance [4,6].

Previous studies have shown that WV reduces organizational performance. This problem requires more investigation and the attention of academicians to identify the possible causes of WV for stakeholders and organizations [7,8]. Individuals who are associated with the health sector of Pakistan face WV and OS that affect their performance. Previously, very few researchers have investigated the direct relationship between WV and OS or WV and SWP, and the relationship between workplace violence, occupational stress, and sustainable work performance is still unexplored. In particular, occupational stress as an intervening construct still needs to be researched. Many workers face OS at their workplace, but they do not disclose their stress due to fear of discrimination, which reduces work motivation among workers and organizations and, in turn, reduces work efficiency. Work performance is being undermined due to OS in the workplace because it creates a bad image that brings toxicity among co-workers and peers [9]. So, this study will be helpful for the health sector of Pakistan to reduce WV and occupational stress and bring sustainability to work performance. It will unveil how workplace violence affects employees’ work life. When violence in the workplace occurs, a robust and balanced work life is converted to a mess.

Based on the above-mentioned research impetus, the purpose of this study is to analyze the relationships between WV, OS, and SWP. WV with multiple dimensions (harassment, mobbing, ostracism, and stalking) was used in this study. Moreover, insights about workplace violence, occupational stress, and sustainable work performance in the literature are discussed. On the basis of the above discussion, the following two research questions (RQ) are addressed:

**RQ1:**  How does workplace violence (harassment, mobbing, ostracism, and stalking) influence sustainable work performance?

**RQ2:**  How does occupational stress intervene between workplace violence (harassment, mobbing, ostracism, and stalking) and sustainable work performance?

## 2. Literature Review and Hypothesis Development

### 2.1. Workplace Violence and Sustainable Work Performance

WV refers to violence, usually in the form of physical abuse or threats, that creates a risk to the health and safety of employees. Ferris, Lian [10] present in their study that WV has four dimensions: harassment, mobbing, ostracism, and stalking. These dimensions are defined as follows: harassment is humiliation and terrorization of one individual by another in the workplace [11]; mobbing means bullying of an individual by a group in any context, such as by family or peers, at school or work, in the neighborhood, community, or online [12]; ostracism is defined as workplace isolation that is perceived by an employee due to his/her peers or employers [13,14,15], with negative consequences for the employee with regard to organizational development in the form of high turnover, lack of work involvement, and high job dissatisfaction [9]; and stalking is a series of actions that make someone feel afraid or in danger. Stalking is a serious crime and can often escalate into violence over time [16]. Sustainable work performance means the coordination of financial, environmental, and social objectives in the delivery of core work activities in order to maximize value [17]. Organizations plan to establish their workforce, including their social nature, in order to improve work performance [18]. WV is a cause of panic and creates an unpleasant environment for workers. This is important for the workplace, where there are teamwork and diverse human resources, and organizations need strong leadership to communicate with workers, stakeholders, and peers [19]. Based on the above discussion, it is understood that WV brings lower levels of organizational commitment and job satisfaction. Moreover, WV creates high levels of depression, anxiety, job burnout, and employee turnover [20].

**Figure 1:**
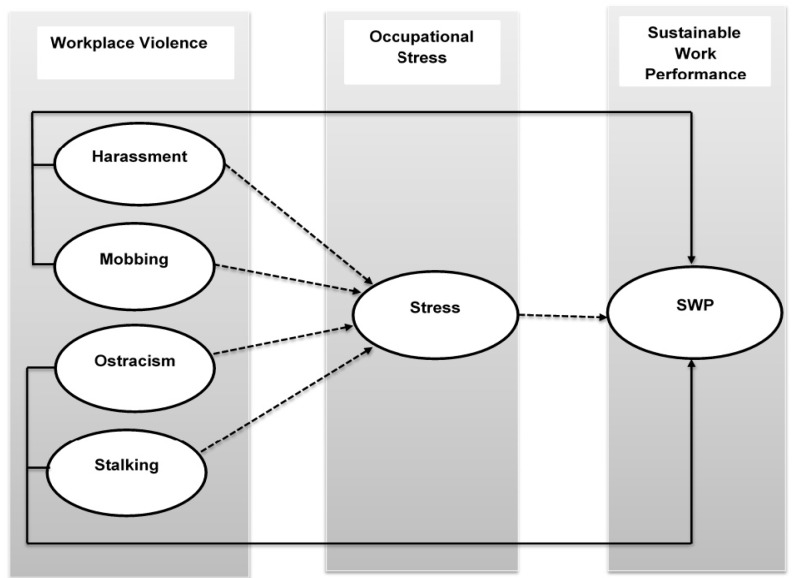
Conceptual model. Arrows indicate negative relationships, dashed arrows indicate indirect relationships, and solid arrows indicate direct relationships. SWP, sustainable work performance.

By breaking down psychological well-being and people’s behaviors in the workplace, WV plays a significant role in damaging workers’ job productivity. First of all, WV threatens psychological resources and their necessity [21]. Having only partial psychological resources is risky for worker development, and to recover such resources, workers need to give more energy, effort, and time [22]. Secondly, WV (harassment, mobbing, ostracism, and stalking) decreases worker morale and unity among co-workers and peers [23]. Due to this serious situation, workers cannot gain access to organizational resources and data since they are taken away from social ties, which eventually results in very low worker performance [24]. Ferris, Lian [10] conducted empirical research on the relationship between WV and sustainable work performance and confirmed that they have a negative relationship [25]. Some past studies showed that WV negatively impacts sustainable organizational performance [17,18]. However, the above-mentioned literature review develops an important understanding of the relationship between WV and SWP. So, on the basis of the above literature, the following hypotheses and below mentioned conceptual model (see Figure 1) were formed:

**Hypothesis** **1a:** Harassment negatively influences sustainable work performance.

**Hypothesis** **1b:** Mobbing negatively influences sustainable work performance.

**Hypothesis** **1c:** Ostracism negatively influences sustainable work performance.

**Hypothesis** **1d:** Stalking negatively influences sustainable work performance.

### 2.2. Mediating Effect of Occupational Stress

Workplace stress is a condition faced by individuals at the workplace in which they are confronted with demands to fulfill that they cannot perceive, so success is out of reach. As a result, their minds become unbalanced. This condition addresses worker stress. One reason for workplace stress is violence in the workplace. WV increases OS among workers and negatively influences sustainable organizational performance. Also, OS has a negative influence in terms of personality disorders among workers. Moreover, OS among workers affects their decision-making ability [26], which will be unfavorable for the organization because of low performance, high turnover, high absenteeism, and enormous economic expense [27]. WV and OS affect the quality of life of workers [28]. Earlier studies showed that 65.3% of Chinese workers faced occupational stress [29]. Frank and Dingle confirmed in their study that occupational stress prompts suicide attempts [9].

WV and OS not only decrease worker productivity and organizational performance, but they also influence the behavior of professionals [30]. Laguna, Mielniczuk [31] argue that workers who experience high work pressure suffer from mental illness and respond with anxiety, aggression, and isolation. Furthermore, work-related pressure and OS negatively intervene in the relationship between WV and job productivity [32]. Thus, as indicated by the above argument, this research shows that OS mediates between WV and SWP. So, on this basis, we propose the following hypotheses and Conceptual model (see Figure 1):

**Hypothesis** **2a:** Occupational stress mediates between harassment and sustainable work performance.

**Hypothesis** **2b:** Occupational stress mediates between mobbing and sustainable work performance.

**Hypothesis** **2c:** Occupational stress mediates between ostracism and sustainable work performance.

**Hypothesis** **2d:** Occupational stress mediates between stalking and sustainable work performance.

## 3. Research Methodology

### 3.1. Research Approach

In this study, we used the questionnaire survey approach. The questionnaire survey is a popular and extensively used research technique for quick collection and analysis of data from the target population [33,34]. The survey analysis approach begins with designing a research instrument [35].

### 3.2. Instrument Development

In this paper, we used WV dimensions (harassment, mobbing, ostracism, and stalking) as independent variables, SWP as the dependent variable, and OS as the mediator. The first section of the instrument describes the purpose of the study and contained instructions for replying, as well as anonymity and privacy statements. The second section of the instrument consists of the respondents’ personal information (gender, experience, position, age, and education). The third part describes the items of the selected variables; 48 items were used with a 5-point Likert scale (1, strongly disagree, to 5, strongly agree). Before data collection, the authors also conducted a pilot study to check the reliability and validity of the questionnaire. We conducted a pilot test of 20 participants (10 academic professors, 10 professional managers) with similar demographics as the final data to check the basis of analysis. The respondents of the pilot study were familiar with the research topic and suggested some modifications to the questionnaire, so revisions were made based on their feedback. The revised questionnaire was distributed for data collection.

### 3.3. Variable Measures

The items of harassment were adopted from Rasool, Maqbool [4]. A total of 10 items were used for harassment, with a 5-point Likert scale (1, strongly disagree, to 5, strongly agree). Sample items included “My supervisor/co-worker/subordinate threatened to fire me from the job if I did not have a romantic relationship with him or her” and “My supervisor/co-worker/subordinate put his/her hand on my back or shoulder while working.” The alpha for harassment was 0.908 (see Table 2) and the standard alpha value is 0.70 or higher, so in this study, the measure was considered adequate.

The items for mobbing were adopted from a previous study by Vveinhardt and Streimikiene [36]. For the measurement of mobbing, 8 items were used with a 5-point Likert scale (1, strongly disagree, to 5, strongly agree). Sample items included “My supervisor/co-worker/subordinate spread gossip and rumors about me” and “My supervisor/co-worker/subordinate had critical areas of responsibility removed or replaced with more trivial or unpleasant tasks.” The alpha for mobbing was 0.912 (see Table 2) and the standard alpha value is 0.70 or higher, so this value met the threshold criteria.

Ostracism used 10 items developed by Rasool, Maqbool [4]. All items were measured with a 5-point Likert scale (1, strongly disagree, to 5, strongly agree). Sample items included “My supervisor/co-worker/subordinate left the area when I entered” and “My supervisor/co-worker/subordinate avoided me at work.” The alpha for ostracism was 0.904 (see Table 4) and the standard alpha value is 0.70 or higher, so the research instrument used for data collection was valid.

Stalking used 9 items developed by Acquadro Maran and Varetto [37]. All items were measured on a 5-point Likert scale (1, strongly disagree, to 5, strongly agree). Sample items included “My supervisor/co-worker/subordinate shouted at me during the conversation” and “My supervisor/co-worker/subordinate at work did not invite me or ask me if I wanted anything when they went out for a coffee break.” The alpha for stalking was 0.834 (see Table 2) and the standard alpha value is 0.70 or higher, so this value met the threshold criteria, which shows that the instrument we used in this study was valid.

Occupational stress used 8 items developed by Hsieh and Tsai [38]. All items were measured on a 5-point Likert scale (1, strongly disagree, to 5, strongly agree). Sample items included “I frequently feel exhausted due to my heavy workload,” “I feel unimportant within my department,” and “I feel incompetent at my current job.” The alpha for occupational stress was 0.949 (see Table 2) and the standard alpha value is 0.70 or higher, so the research instrument we used for data collection was valid.

Sustainable work performance used 10 items developed by Rasool, Samma [39]. All items were measured on a 5-point Likert scale (1, strongly disagree, to 5, strongly agree). Sample items included “I feel that my tasks are more challenging than my co-workers’” and “During the past six months, my actual performance at work is decreasing day by day.” The alpha for sustainable work performance was 0.872 (see Table 2) and the standard alpha value is 0.70 or higher, so in this study, the measure was considered adequate.

### 3.4. Target Population and Sample Characteristics

This was a quantitative study intended to identify the vital and pertinent success categories of a hospital industry in an emerging country. The respondents have been chosen using the convenience sampling method. There are two main reasons to select convenience sampling. First, it is easy to use. Second, in a pilot study, convenience sampling usually used because it allows the researcher to obtain necessary data and trends regarding their study without the complications of using a randomized sample. The target population of this study consisted of Pakistani hospital personnel (doctors, nurses, and paramedical staff). While designing the instrument, we conducted a pilot study. Data were collected from 15 hospitals in the vicinity of Karachi, Lahore, and Islamabad. We distributed 500 questionnaires among the targeted population and received 360 questionnaires, and 15 questionnaires were incomplete. The complete sample size was 345, resulting in a response rate of 69%. The details of the demographics of this research are presented in Table 1.

## 4. Analysis and Results

### 4.1. Confirmatory Factor Analysis

In this study, the model was measured by confirmatory factor analysis (CFA), a method of data analysis that is related to structural equation modeling (SEM). CFA was conducted to judge the convergent and discriminant validity of each construct and to determine the fitness of the overall measurement model. The model enhancement was active to improve appropriate to proposed levels. We excluded some items, and numerous trials were performed to reach the proposed scale levels. As suggested by Hair, Risher [40] the ideal value for data reliability is more than 0.70, so all constructs were measured according to the standard value. Table 2 shows the alpha values of variables, which are all greater than 0.70, and the average variance extracted (AVE) values, which are all greater than 0.50. The factor loading of all constructs is over the threshold value of 0.60.

Table 3 shows the details of the CFA and final models. The details of the discriminant validity are presented in Table 4. The discriminant validity of the constructs was estimated following the instructions of previous studies by Heeringa, West [34], and Jones [41]. The AVE value of all observed constructs was found to be higher than the maximum shared square variance (MSV) and average shared square variance (ASV) values. Similarly, the square root of AVE for each construct is higher than the correlation value, which significantly highlights the discriminant validity of the measurement model.

### 4.2. Descriptive Statistics

Descriptive statistics of the survey respondents are presented in Table 5. The responses were given on a 5-point Likert scale. The mean value of all responses is in the range of 3.42 to 4.05. The standard deviation falls within 0.945 to 1.262.

### 4.3. Regression Analysis

For regression analysis, PLS-SEM 3.2 statistical software was used to analyze the relationships drawn in the conceptual model [42]. PLS is a variance-based structural equation modeling (VB-SEM) approach that enables the simultaneous appraisal of the measurement model (assessing the reliability and validity of the measures of conceptual variables) and the structural model (analyzing the structural links hypothesized between constructs comprising the model) [43,44]. Table 6 shows the results of the direct effects of the four constructs of WV (harassment, mobbing, ostracism, and stalking) on SWP, and the indirect effect of OS between elements of WV environments and sustainable work performance. Table 6 shows that harassment has a significantly negative relationship with SWP (β = −0.824, *p* < 0.05), which supports hypothesis H1a. Similarly, mobbing has a significantly negative relationship with SWP (β = −0.624, *p* < 0.05), which supports hypothesis H1b. Workplace ostracism also has a significantly negative relationship with SWP (β = −0.723, *p* < 0.05), which supports hypothesis H1c. Stalking also has a significantly negative relationship with SWP (β = −0.447, *p* < 0.05), which supports hypothesis H1d; this means stalking has a negative impact on SWP. Furthermore, in an indirect relationship, occupational stress mediates between harassment (β = −0.095, *p* < 0.05), mobbing (β = −0.102, *p* < 0.05), ostracism (β = −0.98, *p* < 0.05), and stalking (β = −0.142, *p* < 0.05) and SWP. As shown in Table 6, hypotheses H2a–H2d are supported.

## 5. Discussion

Most of the studies on WV have been conducted in developed countries. In this study, we contribute to the work of academicians and practitioners by conducting research in a developing nation (Pakistan). This study is the first to examine the influence of WV on SWP in the Pakistani health sector. This study also investigates the intervening role of OS in the relationship between WV and SWP.

First, we concentrated on the direct impacts of WV on SWP, and the results show that WV (harassment, mobbing, ostracism, and stalking) negatively influences SWP, which supports our hypotheses, H1a–H1d. Previous studies showed that WV had a negative relationship with SWP [45,46]. Similarly, Rasool et al. conducted a large-scale survey of banking personnel in China and found that WV had a negative relationship with work productivity [4]. Therefore, it seems possible that negative affectivity may be driving the obtained relationships among the variables.

Second, the mediated effect also translates into significant results, which supports hypotheses H2a–H2d of this study. These results support the findings of past studies [47,48]. Correspondingly, Gandi conducted a study of nurses in selected states of Nigeria. The findings of that study support our study and suggest that occupational stress is a significant element in reducing the sustainable work performance of employees [49], so OS at the workplace is costly for organizations [50]. Unhappy or disengaged employees cost companies billions of dollars each year in lost revenues, settlements, and other damages.

In conclusion, professionals in the health sector, in particular, are required to cope with numerous demands: skills demanded for the job (for instance, being able to understand, alter, lead, and control the behavior of individuals and groups); technical demands (for instance, working in teams, learning new technological skills, communicating effectively with patients); and administrative demands (for instance, dealing with patients). These skills reduce workplace violence to achieve more sustainable work performance. Moreover, these demands could naturally result in numerous pressure situations for health sector personnel. Furthermore, high job demands and work pressures affect the health of employees, resulting in problems such as headaches, insomnia, social dysfunction, and depression. So, we offer some suggestions to reduce workplace violence in organizations that could enhance worker performance. First, organizations need to identify the bad employees who are the root cause of the violence, then provide them with training on soft skills, e.g., relationship management, work time and stress management, and personality development [2]. Second, organizations need to communicate with function heads and supervisors that employees are the greatest asset of the organization and give them directions on how to treat them [4].

## 6. Conclusions

These empirical findings have useful implications for researchers and practitioners. This study advances the debate on jointly investigating WV and OS in order to explain SWP. Our results confirm the linkages among WV, OS, and SWP in Pakistan. Our findings show that in the direct relationship, WV negatively influences SWP, and OS mediates between WV (harassment, mobbing, ostracism, and stalking) and SWP. WV and SWP have an indirect relationship.

The conclusions of this study are as follows: First, harassment is firmly embedded and has distressing effects on the emotional well-being of the entire workplace; it reduces employee morale, which consistently lessens employees’ work performance. Second, mobbing at the workplace increases the level of stress, anxiety, depression, irritability, low work engagement, absence of work performance, and work destruction. Third, ostracism at the workplace reduces motivation among workers and organizations, which reduces work efficiency. Work performance is undermined due to stalking at the workplace because it creates a bad image that brings toxicity among colleagues and peers. Fourth, occupational stress is considered to be a stigma among employees who are facing stress at the workplace, so workers who are facing occupational stress hide their mental status at the workplace because most employees have no awareness of the stress.

Moreover, it is noted that occupational stress is not limited; presenteeism concerns also arise among employees. This is another type of low sustainable work performance, which develops when employees come to work but are mentally absent; due to the occupational stress, they cannot efficiently and effectively perform their job. Moreover, with occupational stress, organizations also encounter worker turnover costs. Finally, we can deduce that workplace violence increases the level of occupational stress among employees. Along with workplace violence, organizational culture will also erode an organization by paralyzing its workforce, diminishing its productivity, and stifling creativity and innovation. We can conclude that if employees are happy and healthy, they can be their most productive. So organizations need to construct a culture where employees can be at their best and shine.

## 7. Practical Implications and Limitations

### 7.1. Practical Implications

The findings of this study indicate some practical steps that could reduce workplace violence. First, organizations should organize some healthy activities for their employees (e.g., family fairs, sports events). Second, managers/supervisors need to identify bad employees who are the root cause of violence at the workplace, then provide them with training on soft skills, e.g., relationship management, work time and stress management, and personality development. Third, organizations need to communicate with function heads and supervisors that employees are the greatest asset of the organization and give them directions on how to treat them. Finally, top-level management should encourage a positive workplace environment and interpersonal cooperation among employees. These steps can help to decrease violence in the workplace and bring sustainability to work performance. Furthermore, these steps could also decrease insomnia, social dysfunction, headaches, depression, and occupational stress among employees.

### 7.2. Limitations

This study also has limitations that affect the interpretation of its results. First, respondents came from a single country (Pakistan), which might imply a cultural bias and limit the generalizability of the conclusions. To further validate the findings, empirical evidence in other cultural contexts is needed. Second, the data were collected from doctors, nurses, and paramedical staff, which might cause a common method bias. Due to differences in the job nature of target populations, future studies should focus on individuals who have similar jobs. In terms of future research, it would be interesting to explore the connection between WV and work productivity using occupational burnout or occupational well-being as a mediating variable.

## Figures and Tables

**Table 1:** Sample characteristics.

Measure	Items	Frequency (*n*)	Percentage (%)
Gender	Male	182	53
	Female	163	47
Experience	Less than 5 years	125	36
	5–10 years	117	34
	More than 10 years	103	30
Position	Doctor	108	31
	Nurse	117	34
	Paramedical staff	120	35
Age	18–25 years	103	30
	25–34 years	107	31
	35–44 years	75	22
	Older than 44 years	60	17
Education	Undergraduate	173	50
	Graduate	103	30
	Postgraduate	69	20

**Table 2:** Construct reliability and validity.

Variable	SA	AVE	CR	Alpha
Harassment	0.810	0.819	0.802	0.908
Mobbing	0.814	0.807	0.715	0.912
Ostracism	0.781	0.754	0.818	0.904
Stalking	0.816	0.780	0.721	0.834
Occupational stress	0.735	0.686	0.857	0.949
Sustainable work performance	0.876	0.673	0.597	0.872

Note: SA, standard loading; alpha, Cronbach’s alpha; CR, composite reliability; AVE, average variance extracted.

**Table 3:** Model fitness.

	CFA Model	Final Model
GFI	0.917	0.926
AGFI	0.872	0.886
NFI	0.924	0.927
TLI	0.908	0.925
CFI	0.937	0.941
RMSEA	0.034	0.038

Note: CFA, confirmatory factor analysis; GFI, goodness-of-fit index; AGFI, Adjusted Goodness-of-Fit Index; NFI, normed fit index; TLI, Tucker–Lewis index; CFI, comparative fit index; RMSEA, root mean square error of approximation.

**Table 4:** Construct reliability and validity.

Variable	AVE	MSV	ASV	Har_All	Mob_All	Ost_All	Stk_All	Os_All	Swp_All
Harassment	0.514	0.532	0.214	0.658					
Mobbing	0.640	0.336	0.237	0.382	0.654				
Ostracism	0.613	0.269	0.215	0.425	0.361	0.674			
Stalking	0.512	0.326	0.326	0.416	0.478	0.561	0.712		
Stress	0.592	0.305	0.261	0.396	0.372	0.488	0.521	0.712	
SWP	0.646	0.376	0.272	0.565	0.287	0.541	0.523	0.489	0.694

Note: AVE, average variance extracted; MSV, maximum shared square variance; ASV, average shared square variance; SWP, sustainable work performance.

**Table 5:** Descriptive statistics.

Variable	*N*	Min.	Max.	Mean	Std. Dev.
Harassment	345	1	5	4.02	0.945
Mobbing	345	1	5	3.42	1.262
Ostracism	345	1	5	3.85	0.986
Stalking	345	1	5	3.82	0.978
Stress	345	1	5	3.96	1.025
SWP	345	1	5	4.05	0.965

Note: Alpha, Cronbach’s alpha; CR, composite reliability; AVE, average variance extracted; SWP, sustainable work performance.

**Table 6:** Regression weights.

**Direct Effect**				
**Hypothesis**	**Estimate**	**S.E.**	**C.R.**	**P**
Hypothesis 1a				
SWP ← Harassment	−0.824	0.034	5.146	***
Hypothesis 1b				
SWP ← Mobbing	−0.624	0.121	6.214	***
Hypothesis 1c				
SWP ← Ostracism	−0.723	0.036	3.224	***
Hypothesis 1d				
SWP ← Stalking	−0.447	0.052	4.264	***
**Indirect Effect**				
Hypothesis 2a				
Stress ← Harassment SWP ← Harassment SWP ← Stress	0.272 −0.112 −0.095	0.034 0.026 0.031	5.61 6.126 8.521	*** *** ***
Hypothesis 2b				
Stress ← Mobbing SWP ← Mobbing SWP ← Stress	0.423 −0.162 −0.102	0.078 0.076 0.019	10.347 0.876 0.910	*** *** ***
Hypothesis 2c				
Stress ← Ostracism SWP ← Ostracism SWP ← Stress	0.423 −0.149 −0.098	0.054 0.057 0.032	9.541 1.490 0.474	*** *** ***
Hypothesis 2d				
Stress ← Stalking SWP ← Stalking SWP ← Stress	0.587 −0.054 −0.142	0.072 0.023 0.038	10.132 0.876 4.465	*** *** ***

*** Significant at the 0.05 level. SWP, sustainable work performance.
